# A retrograde approach for liver gene transfer

**DOI:** 10.1016/j.omtm.2022.11.002

**Published:** 2022-11-25

**Authors:** Nicola Brunetti-Pierri, Paul Gissen

**Affiliations:** 1 Telethon Institute of Genetics and Medicine, Pozzuoli, Italy; 2 Department of Translational Medicine, Federico II University, Naples, Italy; 3 Scuola Superiore Meridionale (SSM, School of Advanced Studies), Genomics and Experimental Medicine Program, University of Naples Federico II, Naples, Italy; 4 Genetics and Genomic Medicine Department, Great Ormond Street Institute of Child Health, University College London, London, UK; 5 National Institute of Health Research, Great Ormond Street Biomedical Research Centre, London, UK; 6 Metabolic Medicine Department, Great Ormond Street Hospital for Children NHS Foundation Trust, London, UK

**Keywords:** gene therapy, hydrodynamic injections, hydrodynamic retrograde intrabiliary injection, nonviral vectors, ornithine transcarbamylase deficiency

The development of hydrodynamic injections was based on the pioneering work by Jon Wolff (1956–2020), who discovered that intracellular delivery of nucleic acids can be achieved by injections of large volumes of naked plasmid DNA solutions^1^ into the portal vein, into the inferior vena cava, or into bile ducts.^2^ Later, in the late 1990s, two independent groups showed that fast injections of large volumes of solution with naked plasmid DNA into the tail vein of rodents resulted in gene transfer in various organs, particularly the liver.^3^^,^^4^ Although clinical application of methods based on the principle of the hydrodynamic injections is limited, this type of injections became very popular for delivery of DNA or RNA in rodents to investigate physiology and/or therapeutic activity of specific genes in the context of a whole animal.

**Figure 1:**
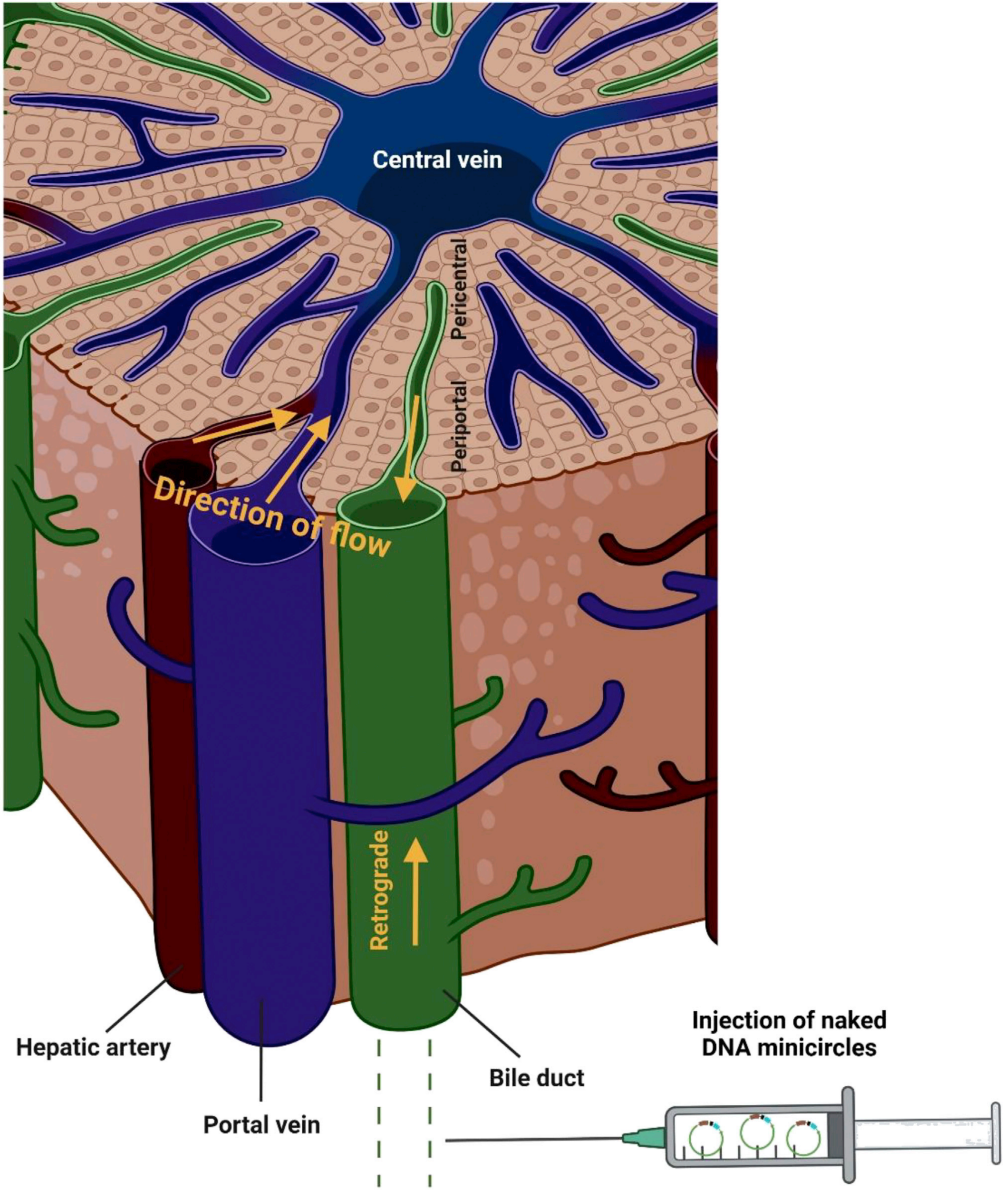
Hepatocytes are arranged in liver lobules that receive oxygenated blood from the heart via the hepatic artery (red) and deoxygenated blood via the portal vein (blue). Bile drains from hepatocytes via the hepatic ducts (green). Hydrodynamic retrograde intrabiliary injection (HRII) is performed by surgical procedure entailing laparotomy and vector delivery via a catheter placed within the common bile duct, which results in retrograde flow through bile ducts and transfection of mostly periportal hepatocytes where OTC is expressed. This figure was created with BioRender.com.

In a study published in *Molecular Therapy* – *Methods & Clinical Development*, Deplazes and colleagues rejuvenated the hydrodynamic intrabiliary injection originally developed by Wolff and applied it to mouse models of inherited metabolic diseases.^5^ Delivery via hydrodynamic retrograde intrabiliary injection (HRII) of DNA minicircles (Figure 1) encoding the *OTC* gene in spf-ash mice, a model of ornithine transcarbamylase (OTC) deficiency, did not result in sufficient therapeutic efficacy, and further optimization is needed. Nevertheless, the method is clinically attractive because it could be performed through endoscopic retrograde cholangiopancreatography (ERCP), a method that has been in clinical practice for many years and was previously tested by the authors in a large-animal model.^6^

The liver is made of hepatocytes operating in repeating units named lobules that are sub-specialized in metabolic processes, a phenomenon termed “liver zonation.” Expression of a given enzyme in its natural layer within the hepatic lobule is expected to be functionally more effective, and more than 50% of liver genes are zonated.^7^ Because of the metabolic zonation along the porto-central axis, gene therapy should aim at expression of therapeutic proteins within the zone where they are physiologically expressed. In contrast to hydrodynamic tail vein injection resulting in gene transfer to pericentral hepatocytes, HRII resulted in targeting of periportal hepatocytes, the cells normally expressing the urea cycle enzymes (Figure 1).

Similarly, adeno-associated virus (AAV) transduction is also not uniform within the hepatic lobule. In mice, for example, AAV8 transduces predominantly hepatocytes near central veins and yields lower transduction levels in hepatocytes in the periportal regions.^8^ In contrast to mice, in nonhuman primates, the expression pattern from AAV8 vectors is reversed, i.e., transgene expression is higher around portal areas and less intense or absent around central veins.^8^ A current gene therapy clinical trial for OTC deficiency with AAV is using AAV8 (ClinicalTrials.gov: NCT05345171), and thus, if the pattern of transduction is similar between nonhuman primates and humans, this approach may not provide expression of the OTC in the cells physiologically expressing the enzyme.

In mice, other AAV serotypes have been found to transduce preferentially periportal hepatocytes^9^ with an opposite pattern compared with AAV8. However, studies in nonhuman primates are still to be performed to establish the transduction pattern of these vectors within the hepatic lobule. The study by Deplazes and colleagues raises the attractive possibility to use the same route of delivery method used for minicircle DNA also for AAV delivery. The advantages of AAV delivery by HRII include the potential of reducing both the dose of vector and toxicity.
